# The effectiveness of EGFR knockdown by RNAi lines varies depending on the tissue

**DOI:** 10.17912/micropub.biology.000612

**Published:** 2022-07-21

**Authors:** Mikaela Follmer, Brandy Shrawder, Kara Eckert, Briana Heinly, Pavithra Vivekanand

**Affiliations:** 1 Susquehanna University

## Abstract

In *Drosophila* , the Epidermal growth factor receptor (EGFR) signaling pathway is known to be critically involved in multiple stages of development. We induced a loss of function phenotype in the eyes, wings, and somatic follicle cells using four EGFR RNAi lines: HMS05003 and JF02283, which produce short hairpin RNAs, as well as JF01368 and KK100051, which produce long hairpin RNAs. Using these four lines, we completed a systematic comparison of the ability of short hairpin vs long hairpin RNAi lines to produce loss-of-function phenotypes in the above-mentioned tissues. Tissue specific knockdown was achieved by using Gal4 drivers specific to the three tissues being studied. In the eyes and wings, the KK100051 line induced the most severe phenotype, while the JF01368 line was the least severe, but in the somatic follicle cells, the KK100051 line was the least effective, while the JF01368 and JF02283 lines were comparable with respect to phenotypic severity. We conclude that there is significant tissue specific variability exhibited by the different RNAi lines.

**Figure 1.**
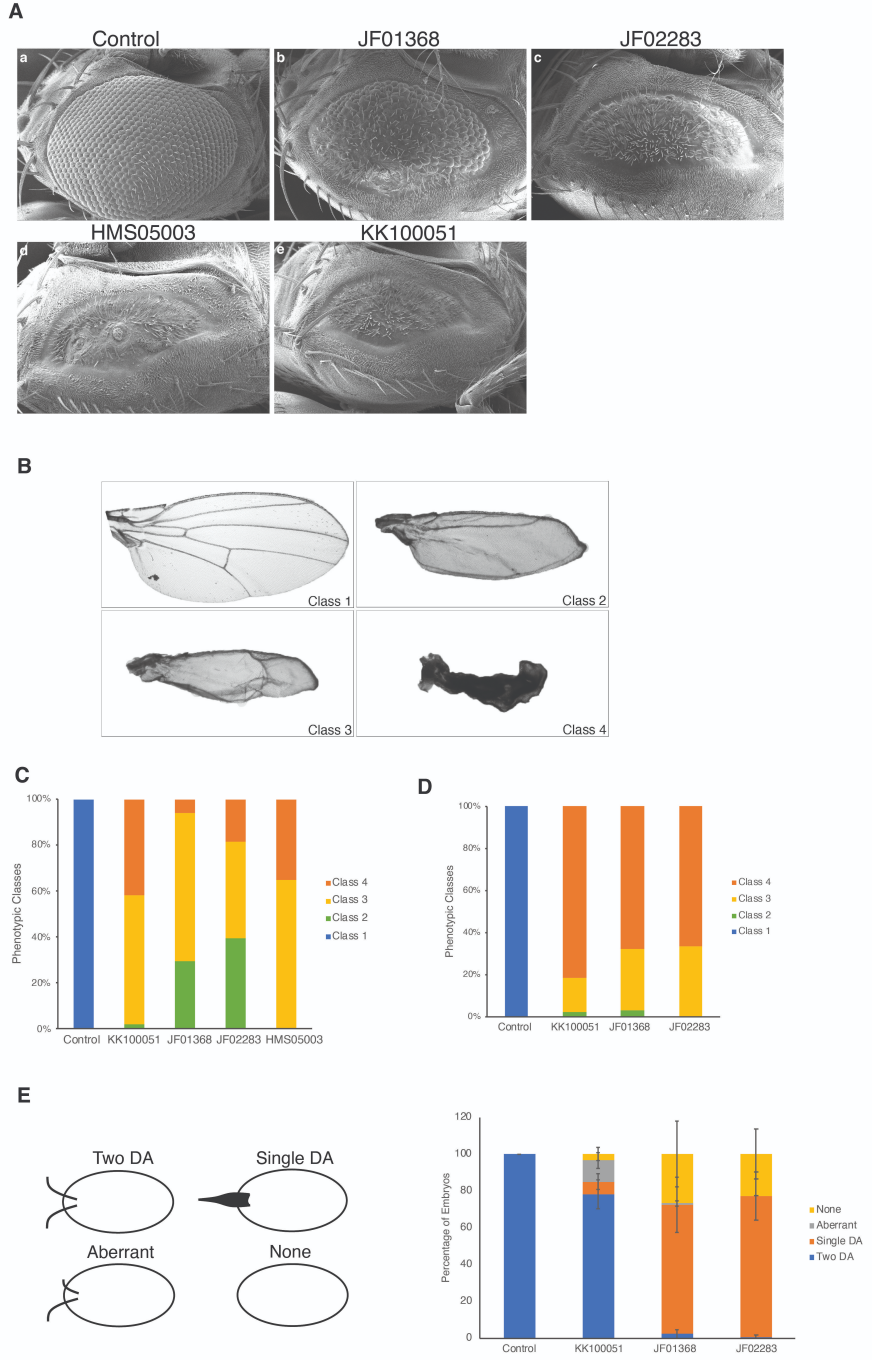
Effect of EGFR RNAi on eye, wing and somatic follicle cell development (A) SEM images of adult eyes showing the phenotype upon knockdown of EGFR RNA using the *logenze-Gal4* driver of (a) control and (b-e) UAS-EGFR RNAi expressing flies. Expression of the JF01368 long hairpin RNA results in the rough eye phenotype with significant reduction in ommatidial number (b). Eye development is progressively affected by expression of the short hairpin RNA from the JF02283 (c), and HMS05003 (d) lines, and the long hairpin RNA from the KK100051 line (e). N= 26, 36, 5 and 2 for JF01368, JF02283, HMS05003, and KK100051, respectively. (B-D) Expression of the long and short hairpin RNAs disrupts vein formation and leads to reduction in wing size. A wing from an adult control fly showing normal formation of longitudinal veins 2-5 (L2-L5) and the anterior (ACV) and posterior cross-veins (PCV). Based on the severity of the phenotypes, the wings were divided into four phenotypic classes. Class 1, wild-type wing; class 2, L2 vein present and reduced wing size; class 3, no veins present and wing size is smaller than a class 2 wing; and class 4, dramatic reduction in wing size. Phenotypes of females (C) and males (D) expressing the different EGFR RNAi lines. No viable males were obtained for the HMS05003 line. Females: n= 48 for KK100051, n= 34 for JF01368, n= 20 for HMS05003, and n= 38 for JF02283. Males: n= 49 for KK100051, n= 34 for JF01368, n= 0 for HMS05003, and n= 27 for JF02283. (E) Eggshells of control embryos have two dorsal appendages (DA), while expression of the different RNAi lines leads to a range of phenotypes, from single dorsal appendage to complete absence of dorsal appendages. Expression of the JF01368 and JF02283 produce similar phenotypic severity with a majority having a single appendage. Majority of the eggshells appear normal with two DA, when the KK100051 line is used to knockdown EGFR.

## Description

RNA interference (RNAi) has been employed extensively in *Drosophila melanogaster* , as a means to investigate the function of either novel genes or to uncover novel roles of previously characterized genes in different developmental processes. The ability to induce tissue specific knockdown has been especially valuable given that several genes such as those involved in evolutionarily conserved signaling pathways are reiteratively used during embryonic and post-embryonic development. This has resulted in a concerted effort to generate different transgenic RNAi library lines with the objective of targeting the entire *Drosophila* genome (Dietzl et al. 2007; Ni et al., 2011; Perkins et al., 2015). These transgenic RNAi lines produce either long or short hairpin double-stranded RNA that results in degradation of the targeted endogenous mRNA (Dietzl et al. 2007; Ni et al. 2008; Ni et al. 2011; Perkins et al. 2015).

Numerous genome-wide *in vivo* RNAi screens have been successfully performed using the different transgenic RNAi collections, and there have been some direct comparisons of the ability of long versus short hairpin RNAs to produce phenotypes (Haley et al. 2008; Ni et al. 2011). However, as a systematic examination of how the same RNAi lines behaved in different tissues had not been undertaken, we had previously examined this issue by knocking down Pointed, an ETS family transcription factor using three different RNAi lines (Bartoletti et al. 2017). We observed that the short hairpin RNA produced the most severe phenotypes in all tissues examined and that long hairpin RNAs were largely ineffective in eliciting a phenotype in embryos (Bartoletti et al. 2017). We were therefore interested in extending this analysis by examining the effect of using different RNAi lines to target the EGF Receptor Tyrosine Kinase (EGFR), which is an upstream regulator of Pointed. We used two long hairpin and two short hairpin RNAi lines to knockdown EGFR in the eyes, wing and somatic follicle cells. We demonstrate that there is a significant degree of variability in the ability of these different RNAi lines to generate phenotypes in the tissues examined. While the long hairpin KK100051 line elicited strong loss-of-function phenotypes when expressed in the eye and wing tissues, its ability to produce phenotypes was markedly reduced when expressed in somatic follicle cells.

Signaling via the EGFR pathway is required for regulating proliferation, spacing, differentiation, and survival in the eye (Dominguez, et al. 1998; Freeman, 1996; Yang and Baker, 2003). Given the myriad roles of EGFR during *Drosophila* eye development, we first examined the effect of RNAi expression in this tissue. The *logenze-Gal4* driver was used to drive RNAi expression in the subset of eye cells, the photoreceptors R1, R6, and R7 and the lens secreting cone cells (Daga et al.1996; Flores et al.1998). We investigated the ability of four different RNAi lines to knockdown the EGFR transcript and examined the phenotype of the adult eyes. Three of the lines were generated by the Transgenic RNAi Project (TRiP) using the VALIUM 10 or the VALIUM 20 vectors. JF01368 was generated using the VALIUM 10 vector and produces a 443 bp product, while the HMS05003 and JF02283 lines were generated using the VALIUM 20 vector, and as a result produce short hairpins of 21 bps. KK100051 is the second of the long hairpin lines that we tested. It is part of the Vienna Drosophila Resource Center (KK100051) RNAi collection and produces a 765 bp product. The region of the EGFR transcript targeted by the different RNAi lines is available by searching the UP-TORR online database ( https://www.flyrnai.org/up-torr/ Flybase Release 6.38). Expression of the long hairpin RNA using the JF01368 line led to a rough eye phenotype with a significant reduction in ommatidial number and the eye field (Fig. 1A,b). Expression of the short hairpin RNA driven by the JF02283 line led to further perturbation of eye development with very few ommatidia developing (Fig. 1A,c). Expression of both the KK100051 and the HMS05003 lines led to lethality with very few flies eclosing from the pupal case. In addition, both lines produced indistinguishable effects on eye development with a complete absence of ommatidial development and reduction in the size of the eye field (Fig 1A, d and e).

During larval development, activation of the EGFR signaling pathway is initiated by binding of the ligand vein and is required for growth and patterning of the wing, as well as the notum (Simcox, 1997; Wang et al. 2000; Zecca and Struhl, 2002a, 2002b). Additionally, the formation of wing veins is promoted by EGFR signaling (Guichard et al. 1999; Sotillos and De Celis, 2005). We therefore wanted to determine whether the four RNAi lines would produce similar degrees of phenotypic severity as was observed in the eye. EGFR knockdown was achieved in the dorsal wing disc by use of the *MS0196-Gal4* driver. Wings from male flies typically displayed a more severe phenotype than wings from female flies. But in both sexes the phenotype ranged from loss of veins to a significant reduction in overall wing size. Since a range of phenotypes were observed, the wings were classified into four categories, based on the size of the wing and the presence or absence of veins, with class 1 being considered a normal wing and class 4 being a rudimentary wing (Fig 1 B). For both males and females, class 1 wings were never observed for any of the RNAi lines. In female flies, expression of the long hairpin RNA by the JF01368 line produced the least severe phenotypes, with class 3 being the largest category observed with 65%, and only 6% of class 4 wings (Fig. 1C). JF02283 was the next with respect to severity of phenotypes produced, with 39% of class 2, 42% of class 3 and 18% of class 4 wings. Interestingly, while no class 2 wings were observed upon expression of HMS05003, 65% and 35% of wings were designated as class 3 and 4, respectively. Lastly, expression of the long hairpin using the KK100051 line resulted in 56% of class 3 wings and 42% of class 4 wings (Fig. 1C). In the males, very few class 2 wings were obtained for all RNAi lines. Additionally, the severity of the phenotypes produced by the JF01368 line was comparable to that produced by the JF02283 line. While expression of the JF01368 line produced 29% of class 3 and 68% of class 4 wings, the expression of JF02283 led to 33% and 67% of class 3 and 4 wings, respectively. Expression of the long hairpin RNA using the KK100051 line, led to 16% and 82% of class 3 and 4 wings, respectively (Fig. 1D). Despite multiple attempts, we were unable to recover any viable male flies upon expression of the short hairpin RNA via the HMS05003 line. Therefore, for both sexes, the KK100051 line produced the most severe phenotypes with 42% and 82% of wings being designated as class 4, for females and males, respectively.

We lastly examined the effect of perturbation of the dorsal-ventral patterning of the follicular epithelium by using the *CY2-Gal4* driver to express the dsRNA in all somatic follicle cells. As expected, 100% of the eggshells of embryos laid by control flies had two dorsal appendages (Fig. 1E). Knockdown of the EGFR transcript resulted in the following three phenotypes to different degrees: the complete absence of dorsal appendages (none), which is indicative of ventralization of the eggshell; a single broad dorsal appendage, which is caused by the conversion of dorsal midline cells to dorsal appendage producing cells (single); and aberrant appendages, where one is significantly shorter than the other (aberrant). Expression of the KK100051 RNAi line, resulted in 78% of the eggshells having 2 dorsal appendages, 11% with single or none and 11% with aberrant dorsal appendages. Expression of the JF01368 long hairpin RNA produced only 2% of eggshells with two dorsal appendages, while 97% of the eggshells had either a single appendage (70%) or none (27%). Similarly, the majority (99%) of the eggshells laid by females in which the JF02283 line was used to knockdown the EGFR mRNA, exhibited either a single appendage (76%) or a complete absence of dorsal appendages (23%) (Fig. 1E). We were unable to recover viable adult females upon expression of the short hairpin RNA using the HMS05003 line. So, in this tissue KK100051 line produced the weakest phenotype.

In the current study, we systematically examined how effectively four different RNAi lines could knock down EGFR, in order to directly compare the efficiency of short versus long hairpin RNA to generate loss-of-function phenotypes in multiple tissues. The JF01368, HMS05003 and JF02283 lines were generated by TRiP, while the KK100051 line was generated by VDRC. Of these four EGFR RNAi lines, only the KK100051 line is predicted to have off-target effects, with two of the 19-mers targeting CG3725 (VDRC: https://stockcenter.vdrc.at/control/main r6.01, and UP-TORR: https://www.flyrnai.org/up-torr/UptorrFly.jsp Flybase Release 6.38). We show that while ommatidial development was impacted by all four RNAi lines, the JF01368 line produced the weakest loss-of-function phenotype with an average reduction in ommatidial number to 150, while the KK100051 and HMS05003 were the strongest and produced comparable phenotypes with a complete absence of ommatidia. We also demonstrate that while the expression of all four RNAi lines impaired wing development with both vein formation and wing size being detrimentally affected, the long hairpin RNA expressed by the KK100051 line elicited the strongest phenotypes in both males and females. In addition, the wings from female flies exhibited weaker phenotypes when compared to the male counterparts from the same RNAi line. This sex difference is not novel, has been observed previously in *Drosophila* , and has been attributed to either differences in developmental timing or to gene sequences that are derived from the X-chromosome that were used to generate the VALIUM vectors (Ni et al., 2008).

Interestingly in both the eyes and wings, though both the JF01368 and the KK100051 lines produce long hairpin RNAs, JF01368 was the weakest while KK100051 was the strongest of the four RNAi lines we tested. A possible explanation for why the phenotypes produced by the VDCR line was considerably stronger could be the length of the hairpin product, since the JF01368 line produces a 443 bp long dsRNA, while the hairpin RNA generated by the VRDC line is 765 bp long. Longer dsRNA’s have been shown to be more effective in gene silencing than shorter dsRNA (Hammond, Bernstein, Beach, & Hannon, 2000; D. Yang et al., 2000). Since the dsRNA generated by the KK100051 line is 322 bp longer than the hairpin produced by the JF01368 line, theoretically more siRNAs might be produced from the VRDC line resulting in enhanced degradation of EGFR mRNA, thereby enhancing its effect in these tissues. However, the KK100051 line produced significantly weaker effects than either the JF01368 or the JF02283 line when expressed in somatic follicle cells. While less than 2% of the eggshells had a wild-type phenotype with two dorsal appendages when either the JF01368 or the JF02283 line was used for EGFR knockdown, 78% of the eggshells exhibited wild-type phenotype when the KK100051 line was used. We observed a similar effect when we used three different RNAi lines to knockdown Pointed in somatic follicle cells, where the VDRC RNAi line was the least effective in producing phenotypes with 80% of the eggshells appearing normal (Bartoletti et al., 2017). The reason for the inability of both the Pointed and the EGFR VDRC RNAi lines to produce strong phenotypes in the somatic follicle is unknown. One possible reason might be that as both of these VDRC lines are inserted into the 30B site on chromosome 2, the expression of the hairpin RNAs is reduced in somatic follicle cells but not in other tissues. Thus, the ability of each RNAi line to elicit loss-of-function phenotypes varied with respect to the tissue in which the EGFR mRNA was being knocked down. Future experiments in which the different RNAi constructs are inserted into the same chromosomal landing site would eliminate the effect of local chromatin effects in different tissues and would provide a more effective comparison of the efficacies of the different targeting constructs.

## Methods

***Fly stocks***

Flies were raised on a standard cornmeal/yeast/agar medium at 25°C. The following strains were used: Canton S; *lozenge-Gal4* (Bloomington Stock # 6313); *MS1096-Gal4* (Bloomington Stock #88600); *CY2-Gal4* (from Trudi Schupbach); HMS05003 = UAS-VALIUM20 EGFR RNAi (Bloomington Stock #60012); JF02283 = UAS-VALIUM20 EGFR RNAi (Bloomington Stock #36770); JF01368 = UAS-VALIUM10 EGFR RNAi (Bloomington Stock #25781); KK100051 = UAS-VDRC EGFR RNAi (KK100051 Stock #107130).

***Scanning electron microscopy***

Adult female flies were dehydrated by incubation in 30%, 50%, 70%, 90%, and two 100% ethanol washes for 10–12 hours each. Following the ethanol dehydration, flies were incubated for 30 min each, in a mixture of 25% HMDS (Electron Microscopy Sciences)+ 75% Ethanol; 50% HMDS+ 50% Ethanol; 75% HMDS+25% Ethanol, and three 100% HMDS. After the third wash in 100% HMDS, the samples were left under a hood to allow the residual HMDS to evaporate. The flies were mounted on carbon adhesive tabs on top of aluminum stubs, and sputter coated with gold using the Denton Vacuum Desk V set to 20 mV for 80s. Images were obtained using the Jeol JSM-6010LV Scanning Electron Microscope. N= 26, 36, 5 and 2 for JF01368, JF02283, HMS05003, and KK100051 respectively.

***Wing Preparation***

Adult flies were dehydrated in 95-100% Ethanol for 24 hours. The ethanol was removed, and flies were air-dried. One wing from each individual was dry-mounted on a slide, and a cover slip was placed over it before imaging. Females: n= 48 for KK100051, n= 34 for JF01368, n= 20 for HMS05003, and n= 38 for JF02283. Males: n= 49 for KK100051, n= 34 for JF01368, n= 0 for HMS05003, and n= 27 for JF02283.

***Analysis of dorsal appendage phenotype of somatic follicle cells***

Virgin females from each RNAi line were crossed to CY2-Gal4 males. Female progeny from the CY2-Gal4 flies crossed to the RNAi lines were mated with Canton S males for 24 hours. The adults were transferred to collection cages with molasses agar plates containing yeast paste. The molasses agar plates were changed every 12 hours. The phenotypes of the embryos were determined and assigned to one of four categories: two dorsal appendages; single appendage; none or aberrant. n= 1126 for Canton S; n= 1467 for KK100051; n= 1137 for JF01368, and n= 913 for JF02283.
